# Designing of Potential Polyvalent Vaccine Model for Respiratory Syncytial Virus by System Level Immunoinformatics Approaches

**DOI:** 10.1155/2021/9940010

**Published:** 2021-05-28

**Authors:** Syeda Tahira Qousain Naqvi, Mamoona Yasmeen, Mehreen Ismail, Syed Aun Muhammad, Syed Nawazish-i-Husain, Amjad Ali, Fahad Munir, QiYu Zhang

**Affiliations:** ^1^Institute of Molecular Biology and Biotechnology, Bahauddin Zakariya University Multan, Pakistan; ^2^University College of Pharmacy, Punjab University Lahore, Pakistan; ^3^ASAB, National University of Sciences and Technology (NUST), Islamabad, Pakistan; ^4^Department of Hepatobiliary Surgery, The First Affiliated Hospital of Wenzhou Medical University, China; ^5^Wenzhou Medical University, Wenzhou, Zhejiang Province, China

## Abstract

**Background:**

Respiratory syncytial virus (RSV) infection is a public health epidemic, leading to around 3 million hospitalization and about 66,000 deaths each year. It is a life-threatening condition exclusive to children with no effective treatment.

**Methods:**

In this study, we used system-level and vaccinomics approaches to design a polyvalent vaccine for RSV, which could stimulate the immune components of the host to manage this infection. Our framework involves data accession, antigenicity and subcellular localization analysis, T cell epitope prediction, proteasomal and conservancy evaluation, host-pathogen-protein interactions, pathway studies, and *in silico* binding affinity analysis.

**Results:**

We found glycoprotein (G), fusion protein (F), and small hydrophobic protein (SH) of RSV as potential vaccine candidates. Of these proteins (G, F, and SH), we found 9 epitopes for multiple alleles of MHC classes I and II bear significant binding affinity. These potential epitopes were linked to form a polyvalent construct using AAY, GPGPG linkers, and cholera toxin B adjuvant at N-terminal with a 23.9 kDa molecular weight of 224 amino acid residues. The final construct was a stable, immunogenic, and nonallergenic protein containing cleavage sites, TAP transport efficiency, posttranslation shifts, and CTL epitopes. The molecular docking indicated the optimum binding affinity of RSV polyvalent construct with MHC molecules (-12.49 and -10.48 kcal/mol for MHC classes I and II, respectively). This interaction showed that a polyvalent construct could manage and control this disease.

**Conclusion:**

Our vaccinomics and system-level investigation could be appropriate to trigger the host immune system to prevent RSV infection.

## 1. Introduction

RSV is one of the most virulent agents for virus-related respiratory tract infections in children and immunocompromised adults worldwide [1–4]. It has been studied that one-third of infant deaths with lower respiratory tract are due to acute infection [5, 6], but there are not any approved prophylactic and *vaccine* strategies *available to manage this infection [*7*].* RSV is a single-stranded negative-sensed enveloped RNA virus that belongs to the *Paramyxoviridae* family [4, 8]. Currently, 14 human RSV-A subtype genotypes and approximately 22 subtype B genotypes have been designated [3, 9]. These subtypes are related to the encoding of 11 proteins responsible for infection progression [10]. The proteomic data revealed that NS1 and NS2 are two nonstructural proteins expressed during the replication phase of the infectious cycle [3, 11, 12]. Eight structural proteins are categorized to perform related functions. RSV nucleocapsid contains phosphoprotein (P), nucleoprotein (N), and polymerase (L) proteins involved in viral replication and integration. Similarly, the viral membrane includes small hydrophobic (SH) protein, glycoprotein (G), and fusion glycoprotein (F) as transmembrane surface proteins related to attachment and fusion with host cells [13]. During RSV encounters, epithelial receptors of host cells provide binding sites and help in viral entry to the cells [3]. After attachment, viral glycoproteins play an important role in the activation of host NF-*κ*B through MAPK associated pathways. TLR and CX3CR1, HSPGs, heparin, annexin-II, and caveolin-1 get activated and cause the increased production of chemical mediators and cytokines [14–16].

Vaccinomics, immunoinformatics, and reverse vaccinology are safer and effective approaches as compared to conventional vaccine design methods providing stable and multivalent vaccines capable of inducing specific immune responses [17, 18]. There is no licensed vaccine currently available for RSV infection; therefore, we designed a potential broad-spectrum polyvalent vaccine against different strains of RSV using these techniques. We designed a comprehensive framework involving software, tools, and databases to find potential and strong immunogens. We retrieved proteomic data through NCBI and analyzed the structural and nonstructural proteins with the help of multiple software to screen out candidate proteins. Based on antigenicity, functional annotation, and system-level investigation, we finalized two glycoproteins (F and G) and a small hydrophobic protein (SH). To examine the binding affinity of these antigenic epitopes to MHC molecules, we developed 3D models of these RSV-specific T cell epitopes and conducted molecular docking studies. This study could ensure the immunogenic/prophylactic option to manage RSV infections.

## 2. Material and Methods

### 2.1. Proteomic Data Retrieval

The genomic and proteomic sequences (structural and functional proteins) of RSVNA1 strain subtype A were accessed and retrieved from the NCBI database in a FASTA format [10]. This research was carried out using computer databases and tools, online servers, and software (Table 1) to design a possible polyvalent vaccine for RSV through integrated steps (Supplementary Figure 1).

### 2.2. Antigenic Protein Screening

Based on subcellular localization, molecular weight, and prediction of antigenicity, the vaccine candidates were screened. The subcellular location of RSV proteins associated with the host cell was initially determined by the Virus-mPLoc server and Uniprot database to separate the transmembrane and surface proteins [19, 20]. Using the VaxiJen v2.0 server, the antigenicity of the shortlisted proteins was predicted (threshold level 0.45) [21]. The alignment-independent prediction of antigenic proteins is the basis of this prediction. The molecular weight of these antigenic proteins was calculated by using the Expasy tool. We used the BLASTp option on the NCBI server to verify the sequence similarity with the host to screen the nonhuman homologous proteins as candidate antigens.

### 2.3. Prediction and Immunogenicity Study of T Cell Epitopes

Multiallelic T cell epitopes of selected candidate proteins were predicted by the online tools ProPred and ProPred-I. These servers apply matrix-based T cell epitope prediction for multiple MHC class I and II molecular alleles [22]. In contrast to a vaccine agent targeting just one form of an allelic molecule, multiallelic epitopes have a wide variety of binding choices. Using the IEDB server, we estimated the immunogenicity of MHC class I epitopes. To discover the immunogenicity of a peptide-MHC (pMHC) complex, this method utilizes amino acid properties and their location within the peptide [22].

### 2.4. Proteasomal Evaluation and Epitope Conservation

To study the spectrum and global effects, we studied the conservation of our selected epitopes. The IEDB tool was used to conduct this study involving various RSV strains concerning human infections [23]. To identify the potential immunogenic regions in the selected proteins which T cells perceive to generate an immune response, the prediction of proteasomal cleavage was significant.

### 2.5. Epitope 3D Modelling

The three-dimensional (3D) peptide structure was generated using the PEP-FOLD server [24]. In structural-based research, 3D structures help to explain the topological representation. The epitopic regions and the underlying pattern of amino acids were observed and visualized using I-Tasser [25] and Chimera [26] servers.

### 2.6. Interaction between Protein-Protein and Host-Pathogen

For the formulation of prophylactic strategies, this interaction analysis is important as it helps to predict protein functions and to play a role in the host immune system and its cellular processes [27]. Cytoscape v3.6.0 software [28] developed the protein-protein interaction (PPI) network and analyzed host-pathogen-protein interactions to reveal prophylactic targets [29]. We developed an integrated pathway model based on the host-pathogen PPI network and to understand the physiological function of potential vaccinating proteins.

### 2.7. Development of Polyvalent Vaccine

For the prediction and evaluation of the best binding combination of epitopes [30] on MHC molecules based on a score, the HADDOCK tool was used. The residues have been calculated and refined using C-port modules and the HADDOCK tool Refinement interface [31]. Linkers were used for polyvalent constructs to prevent unintended attachment of epitopes to each other that could cause variations in the arrangement of amino acids and protein functionality [28]. The HLA-I and HLA-II epitopes were linked together by AAY and GPGPG linkers, respectively [18]. The nontoxic portion of the amino acid sequence of cholera toxin (CTB) was bound as an adjuvant to the polyvalent construct N-terminal to the EAAAK linker [25, 32, 33]. The I-TASSER server modeled the polyvalent construct [26, 34]. 3D refining tools [35], Ramachandran plot [36], and QMean server [37] can estimate the consistency and geometric configuration of the model.

### 2.8. Prediction of Physicochemical Properties and Proteasomal Cleavage of the Polyvalent Construct

The physicochemical properties of the vaccine, including instability index, grand average of hydropathicity (GRAVY), molecular weight, theoretical pI (isoelectric point), aliphatic index, and in vivo and in vitro half-life, were investigated using the ProtParam online server (http://web.expasy.org/protparam/) [38]. The immunodominant epitopes of SYCP1 and ACRBP antigen (C1) were used in the multiepitope vaccine [39], and the SARS-CoV-2 multi-epitope vaccine candidate (C2) [40] was used as positive control for comparing the polyvalent construct's properties. For predicting proteasomal processing, the NetChop (http://tools.iedb.org/netchop/) was used [38].

### 2.9. Prediction of Antigenicity, Allergenicity, Cell Localization, and Immune System Response

To predict the antigenicity of the polyvalent constructs, C1 and C2, the Secret-AAR (http://microbiomics.ibt.unam.mx/tools/aar/) and ANTIGENPro (http://scratch.proteomics.ics.uci.edu/) servers were used. To predict the allergenicity of the candidate vaccine, the AllergenFP v.1.0 (http://ddgpharmfac.net/AllergenFP/) and all six methods of the AlgPred (http://www.imtech.res.in/raghava/algpred/) server were used. The BaCelLo (http://gpcr.biocomp.unibo.it/bacello/) was used to determine the vaccine's subcellular localization [38]. The immune system response of the final construct was analyzed using the C-ImmSim 10.1 server [35]. Binding affinities of peptides to MHC class I and II molecules were predicted, and we observed the T cell epitopes from protein sequences restricted to prevalent HLA-A and HLA-B molecules. The cytotoxic T-lymphocyte (CTL) epitopes were evaluated in the final protein construct representing the MHC peptide binding affinity.

### 2.10. Polyvalent Vaccine's Molecular Docking with MHC Molecules and Toll-Like Receptor 3

Molecular Operating Environment (MOE) software based on the root mean square deviation (RMSD) and binding energy (*E*-score) with default parameters is used for *in silico* binding affinity of multipurpose vaccine construction with MHC molecules and Toll-like receptor 3 (TLR3 receptor). MHC proteins and TLR3 receptor active binding sites were analyzed, and interactions between amino acid residues were observed.

### 2.11. Codon Optimization and In Silico Cloning in Escherichia coli

Codon optimization is the process to increase the translational efficiency of foreign genes in host models if the codons of both species are different from each other. Codon optimization of the polyvalent and target model was performed followed by cloning in *silico* approach. Codon of polyvalent was optimized by using the online tool Java Codon Adaptation Tool (JCAT) server (available at http://www.jcat.de/) [41], and the codons were optimized amenable with the extensively used prokaryotic expression vector *E. coli* K12 strain [42]. Available parameters were chosen as a default. Codon adaptation index (CAI) and GC content were evaluated [43]. For restriction and cloning, BamHI and HindIII restriction sites were added at C and N terminals of polyvalent, respectively. Furthermore, the optimized polyvalent was cloned into *E. coli* pET30a (+) vector using the SnapGene tool (http://snapgene.com/).

## 3. Results

### 3.1. Identifying RSV Antigenic Proteins

**Figure 1:**
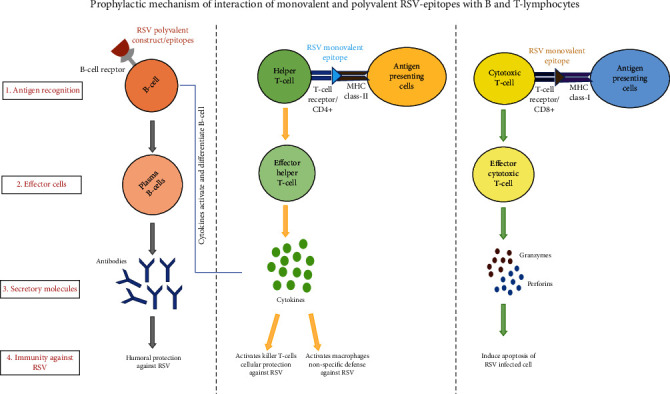
The hypothesis of the interaction of monovalent and polyvalent RSV epitopes with B and T lymphocytes produces the clones of these cells (effector molecules) that protect against RSV infection.

**Figure 2:**
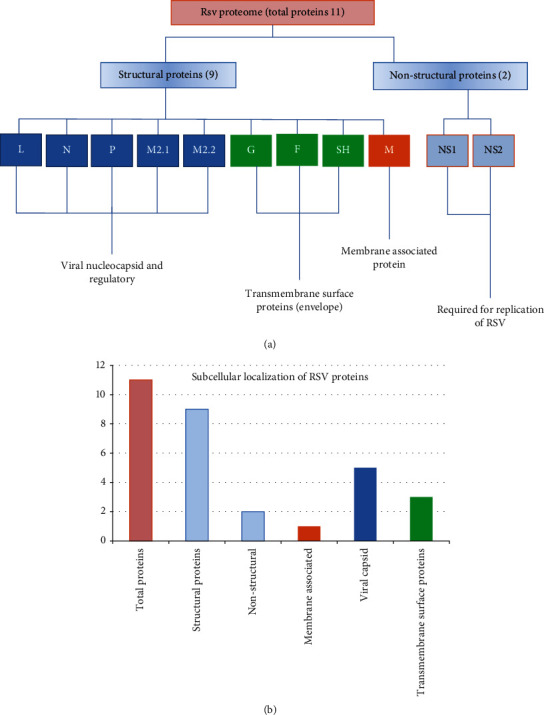
(a) RSV proteome contains a total of 11 proteins out of which 5 proteins have regulatory functions and shape viral capsid (L, P, N, M2-1, and 2), and F, SH, and G encode viral envelopes in transmembrane proteins, whereas M protein is considered to be membrane-associated and two nonstructural proteins, including MS-1 and NS-2, are needed for viral replication. (b) Viral proteome distribution according to subcellular localization.

According to the designed hypothesis of our research, CD8+ cells (cytotoxic T cells) were activated by MHC class I peptides and produce clones of these cells that secreted granzyme and perforin proteins involved in the apoptosis of RSV-infected cells. CD4+ cells were stimulated by MHC class II-specific epitopes that secrete cytokines. These cytokines activate further macrophages and natural killer defense pathways and indirectly antibody production by activating B cells (Figure 1). The strain of RSVNA1 was selected based on an epidemic ratio. Total 11 proteins of the virus were retrieved, among which 9 proteins showed an association with core structure and 2 proteins with functional annotation. As functional proteins were involved in the replication of the virus, the structural proteins act as part of the capsid transacting surfaces and membrane-bound (Figure 2(a)). Subcellular localization prediction was performed to select the secretory/membrane-bounded proteins. The prediction of subcellular localization indicated that 3-envelope transacting proteins existed outside of the host cell while all other proteins localized inside (Figure 2(b)). To deliver the viral material into the host cell, these enveloped proteins were interconnected to bind to receptors of the host cell. Three essential antigenic proteins, namely, G (attachment glycoprotein), F (fusion glycoprotein), and SH (small hydrophobic protein), were found in the antigenicity analysis. The molecular weight was greater than 7.5 kDa (Table 2) of these transmembrane surface proteins, which made them effective antigenic molecules.

### 3.2. T Cell Epitope Prediction

For MHC class I and II molecules, we predicted T cell epitopes of G, F, and SH proteins. These epitopes specific to MHC were multiallelic and can target the human population's multiple MHC alleles. For MHC class I, we predicted four important epitopes and for MHC class II; 5 epitopes were predicted. Each epitope consists of 9-mer residue sequences with important antigenic and immunogenic scores (Tables 2 and 3).

### 3.3. Epitope Conservation and Proteasomal Analysis

**Figure 3:**
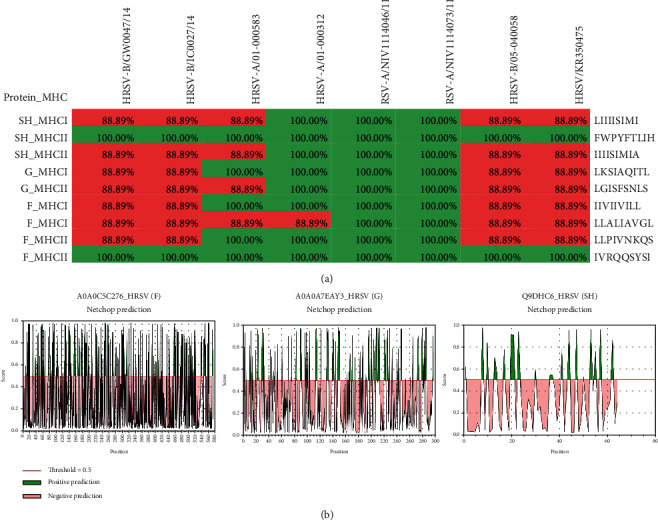
(a) In eight identified strains of RSV, conservancy analysis of potential epitopes. (b) Proteasomal cleavage of candidate proteins indicates significant threshold.

The conservancy study found that these MHC class I and II epitopes had a wide-range impact on eight different strains of human-infected RSV. Two MHC class II epitopes, namely, IVRQQSYSI and FWPYFTLIH, exhibited 100% conservancy in diverse RSV strains, whereas other epitopes are more than 88 percent conserved (Figure 3(a)). For the prediction of proteasomal cleavage that relies on neural network algorithms, we used the NetChop tool [44]. This method was used to describe the C terminal at cleavage sites with a threshold value of 0.5 to classify cleavage and noncleavage sites. Through proteasomes, the cleavage (positive prediction) and noncleavage (negative prediction) sites of antigenic proteins G, F, and SH have been identified to suggest their important function in the presentation of antigen to MHC molecules (Figure 3(b)).

### 3.4. Molecular Modeling of Epitopes

**Figure 4:**
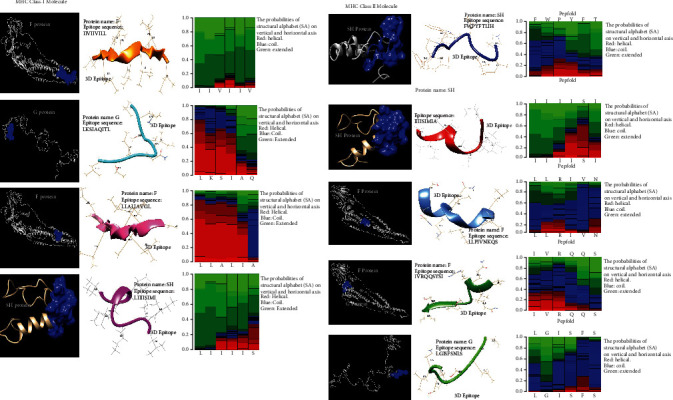
3D models of viral proteins and potential epitopes of MHC classes I and II. Heat maps show the horizontal and vertical axis probabilities of the structural alphabet (SA) (red: helical, green: extended, and blue: coil).

The 3D models describing the structural conformation and configuration of each amino acid residue in alpha-helices were constructed from 9 epitopes of MHC class I and II molecules. The probability of each structural alphabet (SA) on the vertical and horizontal axes is shown in a local structure analysis (Figure 4). In the G, F, and SH proteins, the epitopic area was visualized (blue appearance). The red color shows the helical configuration of the structure in the heat map, green shows expanded, and blue displays coil conformations.

### 3.5. Protein-Protein and Host-Pathogen Interaction

**Figure 5:**
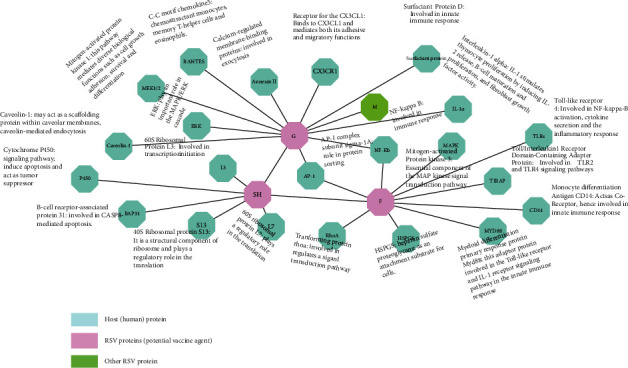
Interatomic host-pathogen study of identified viral candidate proteins with host proteins. In a network, nodes indicate proteins while edges show interactions visualized by different colors.

The protein-protein interaction (PPI) network was designed to assess the topology, functional interaction, and pathological function of RSV proteins with host proteins. High scoring interaction partners (confidence score: >0.9; comprising 26 nodes and 30 edges, nodes show proteins and edges represent interactions) were used in the whole network. This network has been broadly classified into three neighborhoods: pink nodes display the possible antigenic proteins of RSV, and aqua color nodes represent the host (human) proteins that interact with RSV proteins, whereas green nodes are other RSV proteins. The interactive network has shown that RSV proteins interact directly with essential host proteins and are correlated with cell signaling, cell death, aging, endocytosis, immune response (B and T cell receptors), myeloid proliferation, activation of NF-kappa-B, innate immune response, cell growth, activation of transcriptional factors, and migratory activities. Viral G-protein (A0A0A7EAY3, HRSV) interacts primarily with viral M-protein, host ERK-protein (MAPK/ERK cascade), CX3CR1-protein (migratory function), mitogen-activated protein kinase (adhesion, cell growth, differentiation, and cell survival), interleukin-1 (maturation of B cells), NF-kappa-B (immune response), and caveolin-1 (mediate endocytosis). In myeloid differentiation, MAPK kinase signaling cascade, secretion of cytokine, monocyte differentiation, and immune response, SH-protein (Q9DHC6, HRSV) is linked to receptors of B cells, 40S ribosomal translation, cytochrome-P450, interleukin-3, and, likewise, viral F-protein (A0A0C5C276 HRSV) (Figure 5). These antigenic protein associations make them effective in controlling human-related RSV infections.

**Figure 6:**
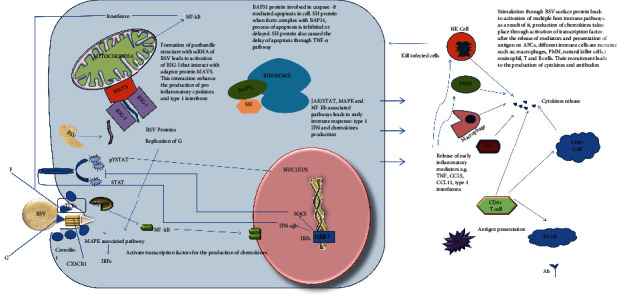
The pathological mechanism of RSV explains the entry of RSV via host cell receptors (CX3CR1 and TLRs) into the host cell activating the immune pathways, including MAPK, JAK-STAT, and TLR-associated pathways, leading to the development of different chemokines and cytokines.

A major functional description and understanding of cellular pathways are provided by this interaction. To initiate the chain of pathological functions, RSV attaches through G and F-proteins to human CX3CR1 receptors. The transcriptional factors and activation of several host immune pathways are impaired by RSV proteins activating MAPK-related pathways and increased chemokine production, JAK/STAT, and NF-Kb associated pathways (Figure 6). The role of our vaccine candidate proteins in disease development and elimination of immune response was identified in a research study about the RSV-host pathway.

### 3.6. RSV Polyvalent Vaccine Construct

**Figure 7:**
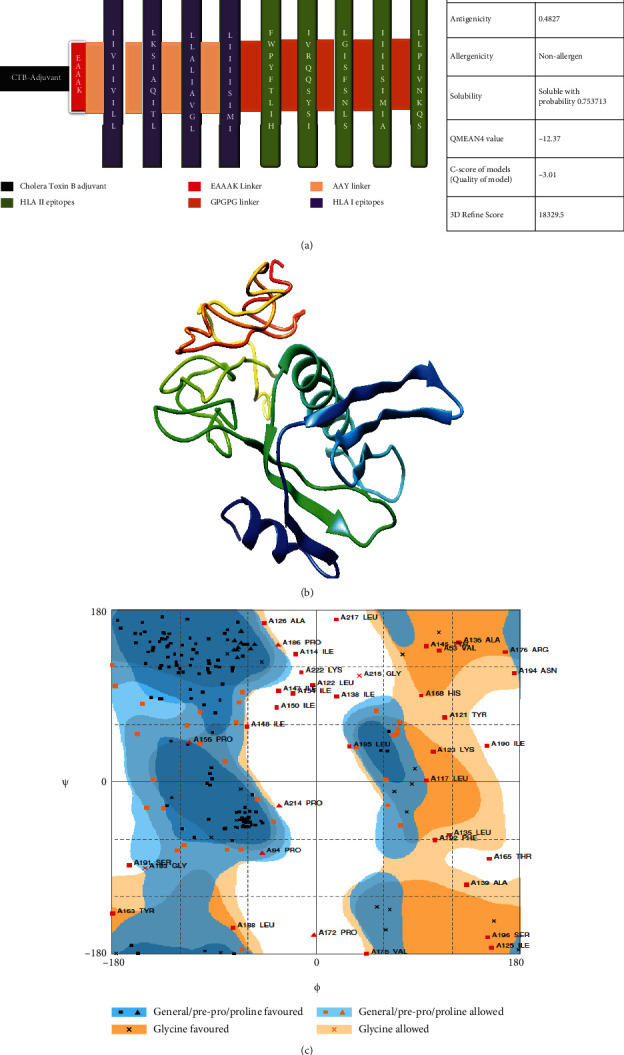
Schematic diagram of polyvalent vaccine construct. (a) 224-long peptide amino acid sequences consisting of an N-terminal adjuvant (black) directly linked to a multiepitope with the help of an EAAAK linker (red). With the help of AAY linkers, MHC I epitopes (purple) were linked, while HTL epitopes (green) were combined with the help of GPGPG linkers. (b) 3D model of the polyvalent construct. (c) Quality assessment of the model by Ramachandran plot indicates 69.4% residues were found in a favored region and 13.5% residues in the allowed region.

The construct of RSV polyvalent consists of 224 amino acid residues including the CTB adjuvant, AAY, EAAAK, and GPGPG linker with a molecular weight of 23.9 kDa (Figure 7(a)) and an optimal antigenicity (>0.45). The tertiary helical conformation and structure are demonstrated by the 3D model of the construct with a confidence score (**C**-score) of -3.011 (Figure 7(b)). 12.6 ± 4.3 Å is the root mean square deviation (RMSD) of the model. The Ramachandran plot displays the low-energy conformations of the model **ϕ** for (phi) and (psi) angles and reflects each residue's local backbone conformation. The majority of residues of the amino acid (57%) are in the favorable and allowed area, suggesting the model's quality (Figure 7(c)).

### 3.7. Physicochemical Properties and Proteasomal Cleavage of the Final Vaccine Construct

The physicochemical properties of peptides have a significant impact on their features such as stability, immunogenicity, and transportation [38]. The ProtParam server was used to quantify the final vaccine construct's physicochemical properties. It has a molecular weight of 23946.27 Da and a pI of 9.04. The ultimate success of a vaccine depends on its stability. The instability index calculates the stability of a protein in a test tube. Proteins with an instability index of less than 40 are considered stable [45]. According to the instability index (II) of 30.26, the polyvalent construct was a stable protein complex. The thermostability of the multiepitope vaccine candidate is represented by the approximate value of the aliphatic index, so as the aliphatic index value increases, the peptide becomes more thermostable [45]. Based on its aliphatic index of 118.13, the final vaccine construct built was a highly thermostable protein. The half-life is a prediction of how long it will take for half of a cell's protein to vanish after it has been synthesized [45]. The physicochemical properties of the final vaccine construct were compared to the positive controls (C1 and C2) indicating high solubility and stability, substantial immunogenicity, bioavailability, and reduced side effects (Table 4). According to the NetChop server's prediction, there were around 65 potential cleavage sites of this construct. In *in vitro* (mammalian reticulocytes cells), *in vivo* yeast, and *Escherichia coli*, the estimated half-life of the final construct was determined to be 30 hours, >20 hours, and >10 hours, respectively, similar to C1 and C2. GRAVY informs us about the hydrophilic or hydrophobic nature of the vaccine construct. The hydrophilic aspect of the peptide is reflected by the negative GRAVY value for the multiepitope peptide vaccine. It is worth noting that peptides with a positive value are hydrophobic [45]. The GRAVY score was 0.438, indicating that the protein is hydrophobic and unable to interact with water molecules in its environment. Solubility is a vital physiochemical property for protein expression and, as a result, manufacturability. Proteins with insufficient hydrophilicity may have a substantial impact on their expression, efficacy, and manufacturability [40]. Furthermore, after overexpression in *E. coli*, the peptide solubility likelihood was 0.753713.

### 3.8. Antigenicity, Allergenicity, Cell Localization, and Immune System Response

**Figure 8:**
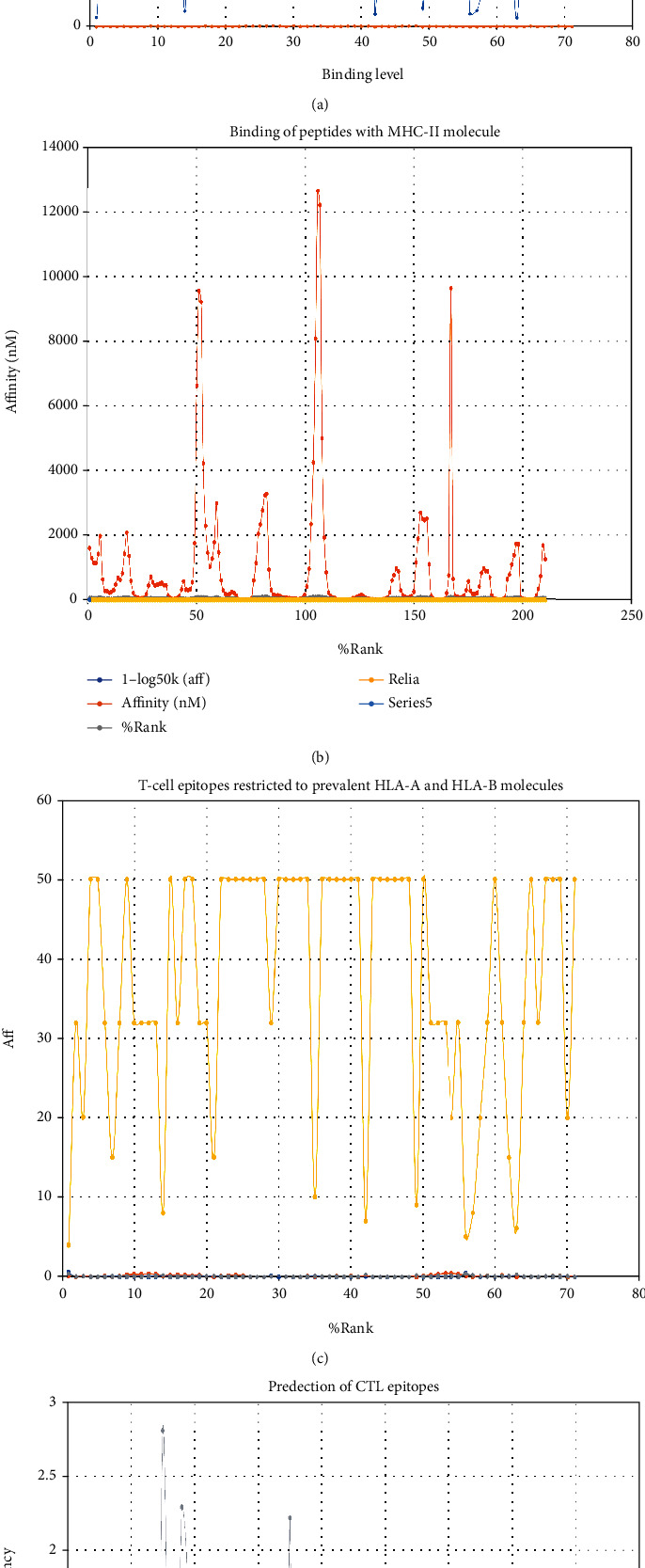
Dynamic simulations of immune response using C-ImmSim 10.1 server and tools. (a) Binding of peptides to MHC class I molecules. (b) Binding of peptides to MHC class II molecules. (c) T cell epitopes restricted to prevalent HLA-A and HLA-B molecules. (d) Prediction of CTL epitopes in protein sequences.

The antigenicity scores of the polyvalent are constructed by Vaxijen 2.0 (threshold value: 0.5), Antigenpro (threshold value: 0.3) [46], and Secret-AAR [40]. Our construct showed an antigenicity score above the threshold value and considered antigenic. A comparative analysis of the antigenicity scores of the polyvalent construct and C1 and C2 has been shown in Table 4. Based on the predictions of the AllergenFP v.1.0 and all approaches of the AlgPred server, the final construct was nonallergenic. Furthermore, the cytoplasm was expected to be the subcellular localization of the vaccine construct and controls. The dynamic simulations of the immune response using C-ImmSim 10.1 tools showed substantial binding affinity of peptides to MHC class I and II molecules involving human MHC alleles HLA-A0201, HLA-DR, HLA-DQ, and HLA-D. There were more than four numbers of high binders, and T cell epitopes were observed from protein sequences. This method integrates three prediction types, peptide MHC binding affinity, peptide-MHC stability, and T cell propensity. T cell epitopes confined to 13 distinct human MHC (HLA) alleles, with 11 of the 12 common supertypes of HLA-A and B. Protein sequences showing the CTL epitopes class I binding for the A26 and B39 supertypes were observed. The server predicts CTL epitopes restricted to 12 MHC class I supertypes. This approach includes the prediction of the MHC class I, proteasomal C-cleavage terminal, and TAP transport efficiency. MHC class I is performed using neural artificial grids for binding and proteasomal cleavage, and the weight matrix is used to analyze the efficiency of the TAP transport (Figure 8).

### 3.9. Molecular Docking of RSV Polyvalent Vaccine with MHC Molecules and TLR3 Receptor

**Figure 9:**
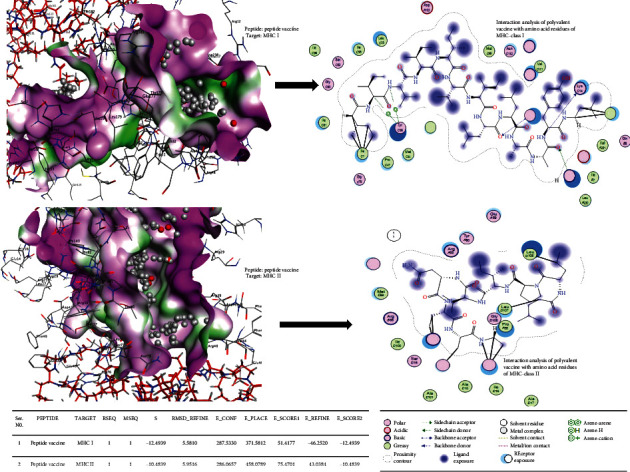
*In silico* binding affinity estimation of the polyvalent construct with MHC molecules by using Molecular Operating Environment software.

Using MOE tools based on binding energy (*E*-score), the in silico binding affinity of the RSV polyvalent construct was evaluated with MHC molecules (class I and II) and TLR3 receptors. MHC molecules and TLR3 receptor binding sites have been evaluated. It was observed that the polyvalent construct shows interaction with SER2, HIS3, SER4, ARG6, PHE8, TYR27, ASP29, ASP30, THR31, GLN32, ARG48, ALA49, PRO50, TRP51, NET98, ASP102, ARG111, TYR113, GLN177, THR178, LEU179, ARG181, THR182, ASP183, TYR209, PRO210, ALA211, GLU232, THR233, ARG234, PRO235, GLY237, ASP238, GLY239, and PHE241 amino acid residues of MHC class-I protein and LYS2, GLU4, ILE8, ASP25, PHE26, ASP27, GLY28, ASP29, ARG76, SER77, THR80, PRO81, ILE82, THR83, ASN84, VAL85, PRO86, LYS111, LEU138, PRO139, ARG140, GLU141, ASP142, HIS143, LEU144, and ASG146 residues of MHC class II molecule. LYS382, PRO408, LEU409, ILE411, LEU412, HIS432, LEU433, GLU434, and VAL435 amino acid residues of TLR3 interacted with the polyvalent construct. By molecular docking of the polyvalent construct with MHC class I, II molecules, and TLR3 targets, the following binding energies were obtained: -12.49, -10.48, and -15.1116 kcal/mole, respectively (Figure 9).

### 3.10. Codon Optimization and E. coli Expression

**Figure 10:**
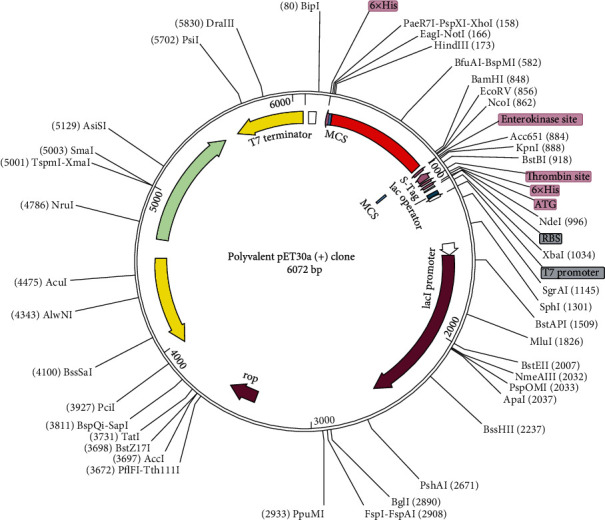
*In silico* cloned codon-optimized polyvalent construct into E. coli strain K12 expression system. The inserted construct DNA sequence is shown in red color, and a cloned construct is of 6072 base-pair length.

*In vitro* cloning was performed to confirm the polyvalent expression in an *E. coli* model. Codon optimization of polyvalent according to *E. coli* strain K12 was performed by the JCAT server. Optimized polyvalent construct contained 669 nucleotides with GC content 50.67% (ideal range of 30%–70%) and CAI value 0.917 (0.8–1.0). The predicted CAI value showed a possible high expression level of polyvalent in the *E. coli* host [47]. Compatible restriction sites HindIII and BamHI were added at N and C terminals of the optimized polyvalent construct, respectively, and cloned polyvalent between these restriction sites of pET30a (+) vector at multiple cloning sites. The final clone had a total of 6072 bp long (Figure 10).

## 4. Discussion

Vaccination is the most important and safe way to prevent pathogenic diseases globally. A major cause of pneumonia and bronchiolitis in children and elderly people is the respiratory syncytial virus. It is estimated that RSV causes approximately 30 million LRT infections and about 60,000 deaths worldwide per year [30]. This global infection burden can be reduced by safe and effective vaccine development, but unfortunately, no potential vaccine is developed at the present time [48]. In clinical trials, the RSV vaccine landscape has been extended to monoclonal antibodies and 19 vaccine candidates to demonstrate the importance of reducing the burden of disease. These candidates have based on four approaches: (1) live-attenuated, (2) particle-based, (3) vector-based, and (4) subunit vaccines [49]. RSV vaccine design is challenging that started with the discovery of formalin-inactivated RSV-vaccine (FI-RSV). It was tested in many randomized control trials that faced the failure of positive results, and the severity of clinical symptoms appeared compared to control group [50]. Conformational changes were observed due to formalin inactivation that leads to the tragic failure of the FI-RSV vaccine and raises the demand for the development of a safer and effective vaccine for RSV infection [36, 50, 51]. The live-attenuated vaccine was developed using RSV A2 strain under low temperatures. However, clinical trials revealed the genetic instability of the virus along with insufficient attenuation [37, 50]. Late-phase failures in the RSV vaccine trial illustrate gaps in immunological protection knowledge and provide lessons for future development [49].

The current study is based on subunit vaccines in comparison to vaccines having a whole pathogenic entity. Meanwhile, the subunit vaccine is comprised of multiple immunogenic parts of the pathogen; as a result, it may develop a strong immune response with few side effects [52]. Finally, vaccine development is a time-consuming, labor-intensive, and costly process. However, in recent years, the development and advances in immunoinformatics (a branch of bioinformatics dealing with the immune system) have brought several reliable tools and servers that resulted in decreased time and cost requirements as a contract of traditional vaccine development. Still, the development of a successful and effective multipeptide vaccine is a difficult challenge in terms of the choice of suitable antigens, immunogenic epitopes, and an effective delivery system. The design of new vaccines based on reverse vaccinology has been developed to improve the prophylactic strategy and overcome the undesirable effects. This approach reduces the time spent in the long array of experimental procedures to avoid hit and trial sets.

In this study, we have designed an immunoinformatics-based comprehensive framework to synthesize a prophylactic type of polyvalent subunit vaccine to control RSV infections. We selected promiscuous RSV epitopes with a common MHC supertype and Toll-like receptor 3 to provide global utilization. These candidate epitopes (F, G, and SH) were highly conserved among different RSV strains. This strategy not only improves the spectrum of RSV strains but also helps to manage emerging mutations. These epitopes showed an optimum binding affinity to MHC molecules and Toll-like receptor 3.

The protein-protein interaction studies showed the viral affinity towards epithelial receptors of the host. Interaction with HSPGs, TLRs, nuclein, and CD14 receptors activates host-immune pathways such as the MAPK-associated signaling pathway [3]. The viral F protein increases the production of IKK/NF-Kb and activates the AP-1 [14–16]. RSV SH-proteins associated with host L3, L7, BAP31, P-450, and transaldolase-1 to modify the immune response [14, 15]. Attachment of glycoprotein to host CX3CR1, HSPGs, heparin, annexin ii, and caveolin-1 receptors stimulates Th1 chemokine, IL-1*α*, RANTES, and other cytokines resulting in inhibition of JAK-STAT pathway due to SOCS accumulation [14, 53]. The cascade of reactions quantifies interferon, interleukins, and other important antiviral factors that could significantly develop the acquired immunity.

Accordingly, a polyvalent vaccine construct was designed by joining the strongest MHC class I and II epitopes with appropriate adjuvants and linkers. The nontoxic part of cholera toxin B protein sequence of 103 amino acid residues (mature peptide) was used as an adjuvant behaving as antimicrobial and as an immunomodulatory [32]. CTB adjuvant was bonded with an EAAAK linker which increased the firmness and bifunctional catalytic activity [54]. Moreover, the epitope of MHC classes I and II was connected with AAY and GPGPG linkers, respectively, to identify each T epitope and allow proficient dissociation [54, 55]. 224 amino acid residues (including AAY, EAAAK, and GPGPG linkers with CTB adjuvant) with a molecular weight of 23.9 kDa are included in the designed RSV polyvalent construct. The polyvalent construct's estimated half-life was calculated as 7.2 hours *in vitro* in mammalian reticulocytes, >10 hours in *Escherichia coli in vivo*, and >20 hours in yeast, which is in the range already reported in subunit vaccine studies [54, 56–60]. Polyvalent construct is antigenic as its antigenicity score is more than the threshold values 0.3 and 0.45 predicted by AntigenPro and Vaxigen v. 2.0, respectively, and it is nonallergic and does not cause any inflammation or allergic reactions when injected. Molecular docking analysis showed that the predicted construct has a stable connection with the TLR3 receptor with less binding energy, which means it can easily be distributed within the body and provoke the immune system strong. Therefore, our results showed that our predicted polyvalent construct has a strong binding affinity towards immune receptors.

After *in silico* cloning in the expression vector system, the codon adaptation index (CAI) value suggests the translation efficiency of cloning, which means the mRNA codons of polyvalent are compatible with the host system. CAI with higher GC content indicates that synthesis of polyvalent vaccine is possible at the experimental level with greater expression in *E. coli* strain K-12 expression system. This *in silico* cloning was performed by using the pET30a (+) vector to design potential vaccine candidates. For the purification of the product, the pET30a (+) vector has both His- and S-tags that help in the purification of protein. S-tag also increases the stability of a protein product [54]. *In silico* cloning of potential vaccine candidates has already been studied [56]. It is studied that RSV peptides activating multiple alleles provide a solid base for the prediction of vaccine design. These polyvalent peptides can be further experimentally verified for developing effective RSV control.

## 5. Conclusion

We predicted 9 novel epitopes of three RSV proteins (G, F, and SH). These epitopes have the potential ability to provoke the immune system, so it could be an excellent target for peptide vaccines. A significant association of these viral peptides in host signaling pathways and immune responses has been found.

## Figures and Tables

**Table 1:** Databases/tools/servers used for the design of RSV polyvalent vaccines.

Databases/tools	Web link	Purpose	Reference
NCBI	https://www.ncbi.nlm.nih.gov/	Data accession	—
VaxiJen server	http://www.ddg-pharmfac.net/vaxijen/VaxiJen/VaxiJen.html	Antigenicity prediction	[21]
Virus-mPLoc	http://www.csbio.sjtu.edu.cn/bioinf/virus-multi/	Subcellular localization	[19, 20]
HADDOCK 2.2	https://haddock.science.uu.nl/	Epitope docking	[31]
ProPred	http://crdd.osdd.net/raghava/propred/	MHC class II epitope prediction	[60]
Propred1	http://crdd.osdd.net/raghava/propred1/	MHC class I epitope prediction	[22]
IEDB (NetChop)	http://tools.iedb.org/netchop/result/	Proteasomal cleavage analysis	[44]
IEDB	http://tools.iedb.org/conservancy/	Epitope conservational studies	[23]
PEPFOLD	http://bioserv.rpbs.univ-paris-diderot.fr/services/PEP-FOLD/	Epitopes 3D modeling	[24]
Cytoscape	http://www.cytoscape.org/	Protein-protein interaction	[61]
MOE	https://www.chemcomp.com/MOE-Molecular_Operating_Environment.htm	Epitopes affinity prediction	—
ITASSER	https://zhanglab.ccmb.med.umich.edu/I-TASSER/	3D modeling	[26, 62, 63]
Chimera	https://www.cgl.ucsf.edu/chimera/	Protein visualization	[64]
Qmean	https://swissmodel.expasy.org/qmean/	Quality estimation	[65]
Rampage analysis	http://mordred.bioc.cam.ac.uk/~rapper/rampage.php	Confirmation of amino acids	[66]
3D refine	http://sysbio.rnet.missouri.edu/3Drefine/	Protein refinement	[67–69]
Antigen pro	http://scratch.proteomics.ics.uci.edu/explanation.html#ANTIGENpro	Prediction of antigenicity	[70]
VaxiJen	http://www.ddg-pharmfac.net/vaxijen/VaxiJen/VaxiJen.html	Prediction of antigenicity	[21, 71]
AllergenFP	http://www.ddg-pharmfac.net/AllergenFP/	Predicting Allergenicity	—
AlgPred	http://crdd.osdd.net/raghava/algpred/submission.html	Predicting Allergenicity	—
Sol pro	http://scratch.proteomics.ics.uci.edu/explanation.html#SOLpro	Solubility prediction	[70]
PROSO	http://mbiljj45.bio.med.uni-muenchen.de:8888/proso/proso.seam	Prediction of solubility	[72]
Expasy	http://web.expasy.org/compute_pi/	Molecular weight prediction	[73, 74]

**Table 2:** T cell epitopes for MHC class I as potential vaccine candidates indicate significant antigenic scores.

Uniprot_ID	Protein name	Molecular weight (kDa)	Gene symbol	Epitope sequence	Peptide position	No. of alleles	Antigenicity	Immunogenicity
A0A0A7EAY3_HRSV	Attachment glycoprotein	32.69	G	LKSIAQITL	35	4	0.6024	0.1442
A0A0C5C276_HRSV	Fusion glycoprotein	63.28	F	IIVIIVILL	531	16	0.5705	0.4216
—	—		—	LLALIAVGL	538	13	1.4491	0.2226
Q9DHC6_HRSV	Small hydrophobic protein	7.53	SH	LIIIISIMI	11	10	0.6058	0.1607

**Table 3:** T cell epitopes for MHC class II as potential vaccine candidates indicate significant antigenic scores.

Uniprot_ID	Protein name	Molecular weight (kDa)	Gene symbol	Epitope sequence	Peptide position	No. of alleles	Antigenicity
A0A0A7EAY3_HRSV	Attachment glycoprotein	32.69	G	LGISFSNLS	97	22	2.0701
A0A0C5C276_HRSV	Fusion glycoprotein	63.28	F	LLPIVNKQS	203	13	1.4281
—	—	—	—	IVRQQSYSI	280	32	1.3637
Q9DHC6_HRSV	Small hydrophobic protein	7.53	SH	FWPYFTLIH	14	13	1.2475
—	—	—	—	IIIISIMIA	31	46	0.5546

**Table 4:** Comparative analysis of the physicochemical and antigenicity properties of the polyvalent construct and positive controls (C1, C2).

Properties	Parameters/tools	C1	C2	Polyvalent construct
Physicochemical	Molecular weight	6.38 kDa	51.64 kDa	23.9 kDa
	pI value	9.61	10	9.04
	GRAVY	-1.127	-0.354	0.438
	Instability index (II)	33.38	27.09	30.26
	Aliphatic index	53.53	79	118.13

Antigenicity	Vaxijen 2.0	0.61	0.59	0.50
	Secret-AAR	35.4	39.8	37.5
	ANTIGENpro	0.62	0.74	0.4089

## Data Availability

The data was submitted with the article.

## Supplementary Materials

Supplementary Materials Supplementary Figure 1: the designed framework showed the integrated steps involved in target protein screening and antigenic multiepitope mapping in respiratory syncytial virus using a computational approach.
