# Supplementary figures and images for: Designing of Potential Polyvalent Vaccine Model for Respiratory Syncytial Virus by System Level Immunoinformatics Approaches
Source: Biomed Res Int. 2021 May 28;2021:9940010. doi: 10.1155/2021/9940010 (PMC8177976; doi:10.1155/2021/9940010)

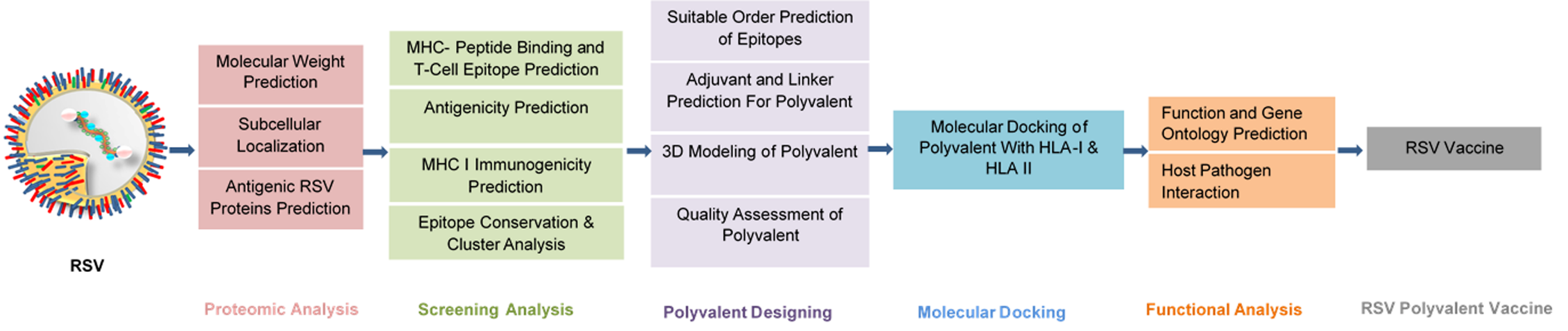


**Supplementary Figure 1**

Supplement: Supplementary Materials — Supplementary Figure 1: the designed framework showed the integrated steps involved in target protein screening and antigenic multiepitope mapping in respiratory syncytial virus using a computational approach. [file 9940010.f1.zip › Supplementary Figure 1.docx]

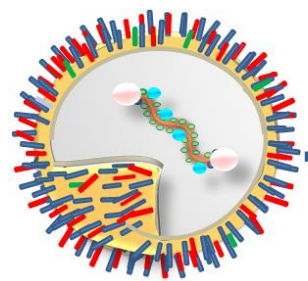

RSV

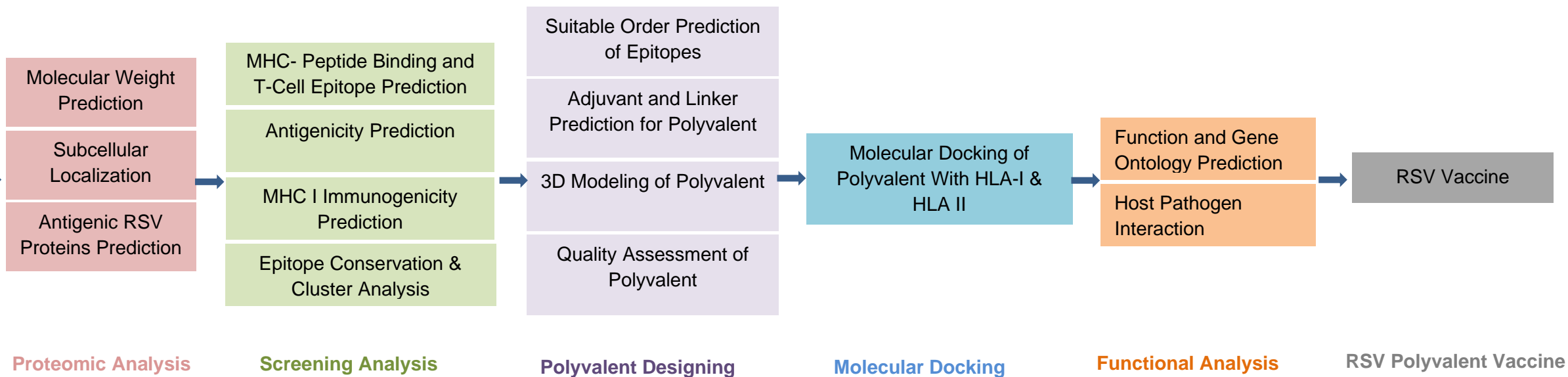

Supplement: Supplementary Materials — Supplementary Figure 1: the designed framework showed the integrated steps involved in target protein screening and antigenic multiepitope mapping in respiratory syncytial virus using a computational approach. [file 9940010.f1.zip › Supplementary Figure 1.pdf]
